# A One-Stage Ensemble Framework Based on Convolutional Autoencoder for Remaining Useful Life Estimation

**DOI:** 10.3390/s22072817

**Published:** 2022-04-06

**Authors:** Yong-Keun Park, Min-Kyung Kim, Jumyung Um

**Affiliations:** Department of Industrial & Management System Engineering, Kyung Hee University, 1732, Deogyeong-daero, Yongin-si 17104, Korea; yongkp@khu.ac.kr (Y.-K.P.); gse06064@khu.ac.kr (M.-K.K.)

**Keywords:** modular factory, Industry 4.0, smart factory, energy-efficient process, deep learning, classification, neural network

## Abstract

As the legislative pressure to reduce energy consumption is increasing, data analysis of power consumption is critical in the production planning of manufacturing facilities. In legacy studies, a machine conducting a single continuous operation has been mainly observed for power estimation. However, the production machine of a modularized line, which conducts complex discrete operations, is more like the actual factory system than an identical simple machine. During the information collection of this kind of production line, it is important to interpret mixed signals from multiple machines to ensure that there is no reduction in the information quality due to noise and signal fusion and discrete events. A data pipeline—from data collection (from different sources) to preprocessing, data conversion, synchronization, and deep learning classification—to estimate the total power use of the future process plan, is proposed herein. The pipeline also establishes an auto-labeled data set of individual operations that contributes to building an power estimation model without manual data preprocessing. The proposed system is applied to a modular factory, connected with machine controllers, using standardized protocols individually and linked to a centralized power monitoring system. Specifically, a robot arm cell was investigated to evaluate the pipeline, with the result of the power profile being synchronized with the robot program.

## 1. Introduction

### 1.1. Background

The field of prognostics and health management (PHM) was initiated in the electronics industry in 2006 [1] and extended to the rotary machinery industry in 2014 [2]. PHM is intended to increase productivity in machine maintenance [3]. Since 2011, Industry 4.0 has spread widely as a tool to build an infrastructure capable of collecting data from devices, machines, and entire systems. PHM has been widely discussed in the literature, and various methods for PHM, including machine learning and deep learning, have been proposed [4].

At the PHM stage, data are first collected using sensors. After the health of the system is determined based on the collected data, an abnormal situation is diagnosed and the remaining useful life (RUL) is predicted. Health indicator (HI) extraction, an indicator of machine health, is another indispensable process, based on which, the time until machine failure can be predicted. Therefore, PHM technology may be serviced only when needed and the optimum maintenance cycle and range for formulating a maintenance strategy can be determined, leading to a cost-reduction effect [4].

Bearings are the main component of analysis for predictive maintenance of machinery. Machines contain several rotating parts, and bearings account for approximately 40% of machine failures [5]—the highest rate of failure among the various components of a machine. Therefore, remaining useful life prediction for bearings is used in the main predictive maintenance method. Bearing RUL prediction is primarily analyzed using vibration signals and sound [6], for which various signal processing technologies, such as noise reduction and fault frequency measurement, are utilized.

### 1.2. Limitation of Legacy Preprocessing and Deep Learning Models

There are several methods to extract features in various frequency domains; however, the use of a single domain is problematic. For example, if only the time domain model is used, then a prediction model skips learning the crack signal which occurred in the frequency spectrum; thus, the model can miss the information that the features have in each domain, and even though some models and machines perform well, the problem resides in that the preprocessing method depends on the data specific to each domain. Therefore, many studies have suggested features created from various domains of time, frequency, and time–frequency in order to complement the shortcomings of aforementioned methods consisting of extracting a single domain feature. However, despite these attempts, there exist drawbacks, since the features of the three domains are lumped into one data set, i.e., simple concatenate, that is used as the input of the model. This method is insufficient to efficiently extract the information included in each domain, and to conduct it as a solution for multi-domain feature extraction.

Selecting the best feature data set or using latent space of AE (autoencoder) have been typical methods of achieving multi-domain feature extraction. Both methods, however, have some drawbacks. Using a particular feature makes a learning model rely on specific metrics. It diminishes the benefits to build a data set from multiple domains. For example, it is worthwhile to add a specific feature capturing the critical moments of the breakage, even though this gives the model a negative influence during most of the learning process. Feature selection ignores features showing the correlation in only a certain moment.

### 1.3. Contributions and Novelty

**Figure 1:**
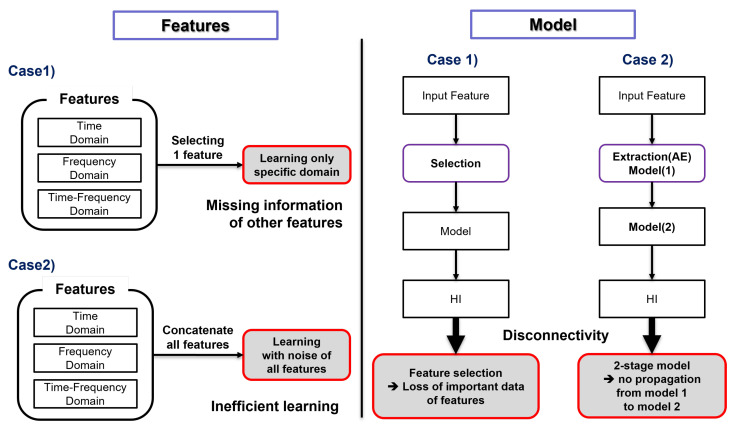
Disadvantages of legacy preprocessing and learning models: (Features) Case 1 is feature selection, while Case 2 is feature concatenation. (Model) Case 1 is feature selection with learning the health indicator. Case 2 is 2-stage learning after feature extraction.

To eliminate the drawbacks of feature selection, several studies have adopted latent space to reduce the dimension using deep learning models, in particular AE, in feature extraction. However, feature extraction through AE has a complexity problem arising from the need to learn two different prediction models: the first is the AE, for learning, and the second is the latent space and RUL for predicting HI. Learning the two models has the disadvantage of increasing the complexity of the model structure, meaning that the efficient learning of the latent space is not performed. The reason for this is that, after learning AE, features are extracted using the latent space. The extracted features are then used to train the RUL prediction model. The disadvantage of this method is that HI, which is the final output value of the RUL prediction model, is learned without any interaction with the features extracted through the latent space. The drawbacks addressed here are illustrated in Figure 1.

In this paper, the authors suggest a method using an all-domain data set in a one-stage learning model, including latent spaces of AE and an RUL model to predict HI. The contributions and novelties of this paper are the following:

1. The legacy learning model has inefficient preprocessing and indirect connection to the health indicator.
2. The proposed method utilizes wavelet decomposition and the latent space of the autoencoder to extract features from different domains.
3. Furthermore, a one-stage learning model is applied to keep the back-propagation from the health indicator model to the preprocessing model.
4. The proposed approach was applied to the PRONOSTIA dataset and showed overall better performance of monotonicity, trendability, and root mean square error.

The remainder of this paper is structured as follows: Section 2 surveys relevant state-of-the-art research. Section 3 describes the proposed model architecture. Section 4 compares the proposed model with relevant models of HI prediction. Section 5 provides the discussion and conclusion.

## 2. The Literature

A survey sensor-data-driven approach is required for the preprocessing of sensor signals, wherein vibration signals are analyzed using various signal processing techniques. Preprocessing is performed on three main domain features: the time domain feature, the frequency domain feature, and the time–frequency domain feature. In the case of time domain features, various statistics have been widely used to efficiently capture bearing failure signals. Typically, statistical features, such as root mean square (RMS) and kurtosis, which describe the overall bearing failure process, are used. However, the main drawback is that these features do not describe the detailed failure steps. In the second case, frequency domain features are mainly analyzed by transforming time domain signals, such as Fourier transform (FT) and Hilbert–Huang transform into the frequency domain. The advantage of these features is that the technology is known to efficiently perform the steps of initial failure and final failure. However, their disadvantage resides in failing to describe the procedure for intermediate failures. Finally, time–frequency domain features are analyzed based on short-time Fourier transform (STFT) and wavelet transformation (WT). These transformation features are known to generate fewer errors and eliminate the loss of information [7].

After the preprocessing is performed, meaningful features are selected, identifying the so-called feature selection method. Feature selection involves selecting a more efficient feature to obtain a bearing failure sign and remove unwanted features. In this process, features are selected based on various metrics, such as monotonicity, trendability, and robustness [8]. In addition to feature selection, the process for selecting sensitive features also consists of feature extraction. Feature extraction is a method for creating new features through the use of existing features. Principal component analysis (PCA), independent component analysis (ICA), partial least squares, and self-organizing map (SOM) are some of the methods used for this purpose [9]. In addition, a feature extraction method, through deep learning with a model or neural network, extracts its own function using a convolutional neural network (CNN) or autoencoder (AE). AE has attracted considerable attention as a feature extraction method that utilizes latent space [8,9,10].

RUL prediction has been extensively studied, but two general methods are widely used. The first method involves predicting the change of breakage through a physical model [11], and the second involves predicting degradation based on sensor data [12]. The physical model is practically difficult to implement as an actual complex facility in an operating environment. In other words, expressing different situations while keeping few failure mechanisms is bound to be limited. For this reason, machine learning techniques that learn from sensor data based on a black-box model are widely applied [13]. The comparison of related papers is shown in Table 1.

### 2.1. Domain Selection

In many studies, various features have been used in three domains to analyze the bearing vibration signal: time domain, frequency domain, and time–frequency domain. Using the time domain as an input feature consists of applying a set of statistical factors extracted from time series signal data. Fundamentally, RMS has been used in bearing diagnosis and HI [22]. Adding the window size in RMS is suggested in order to depict the changes with time [23]. Among the statistics used as features in the time domain [24], a statistic called waveform entropy has been reported. After decomposing signals into specific levels using wavelet packet decomposition (WPD), 14 statistics were extracted for each decomposed signal level [25]. In addition, many studies have been conducted using the original signal [26]. The window size was adjusted and the original signal was used as an input. There have also been attempts to find meaningful information only from the original signal through CNN [16].

A previous study [27] constructed input data by using the frequency signal applying a moving average after FT application. Other studies [24,28] have focused their research using FT as input data.

While some studies have used the time–frequency domain, other studies proposed the creation of an input shape similar to an image in a two-dimensional format, using the STFT and learning through CNN [17]. In addition, a study [15] imaged every signal using WT and then extracted HI using a CNN network.

There also exist studies using a multi-domain input. These studies [7,14,29] obtain features using statistics as a time domain feature, FT as a frequency domain feature, and WT as a time–frequency domain feature. One study [20] obtained features in three domains, reducing the dimension through restricted Boltzmann machine (RBM), and predicting HI through gated recurrent units (GRU). Another study [19] did not use all three domains, resorting to statistics and raw signals in some cases, whereas two other studies [30,31] used a relevance vector machine (RVM) to construct a model, and predicted HI using statistics and a frequency domain feature, respectively.

### 2.2. Feature Selection and Feature Extraction

After collecting a feature in each area, both feature selection and extraction remove unnecessary signals and keep only useful information. Various metrics such as monotonicity, trendability, and robustness are used for feature selection. In [24], only the trendability feature applies to machine learning. In other machine learning techniques, one study [14] selected three metrics: trendability, monotonicity, and the average of these two metrics, while another [25] used the Mahalanobis distance of the features. One study [32] highlighted the fact that there have also been studies selecting features by using the information theory metric. These studies used statistical techniques as feature selection. Sensitive features using the chi-square test [33] and F-test [34] were applied to feature selection.

Following feature selection, AE is often performed for feature extraction. Lin and Tao extracted features using a latent space after ensembling a number of AEs and used it as an input to a model that predicts RUL [18]. Similar studies used three domains as conducted feature sets, whereas Ren et al. used latent space and the remaining set of domain features as inputs for the RUL prediction model after feature extraction via AE only in the time domain [7]. Xu et al. also applied the AE model to only predict the final output value of HI [27]. AE is a deep learning model with equivalent input and output; however, the input data is not restored. Similarly, there is a PHM study that does not restore input data [35], the difference being that a network is designed for bearing failure classification from the final output value. Some studies attempted to conduct both feature selection and extraction simultaneously. Hu et al. and She et al. performed feature extraction through RBM and then selected features based on the HI metric [29,31].

### 2.3. Deep Learning Model of Remaining Useful Life

After extracting the features for bearing failure of the vibration signal in various domains, the remaining useful life is predicted via a deep learning model. Deep running models typically used multi-layer perceptron (MLP), recurrent neural network (RNN), and CNN [10]. Using these models, the remaining useful life was efficiently predicted through the features extracted. After demonstrating the efficiency of the latent space using AE, AE was used to reduce the dimensions and perform feature extraction. The features extracted in this manner were used as inputs to the model for predicting the remaining useful life, creating a bearing prediction model with an even higher performance [12].

### 2.4. Summary and Opportunities

Many previous studies have used a specific domain to extract features. There are cases where a single domain is used as a feature and other cases where two or more domains are used. However, a common point to both types of cases is that feature extraction was simply made into one dataset and used as an input to the model. The problem in using only one domain as a feature is that characteristics of the remaining features are missed. This issue arises even when more than one domain is used as a feature. It simply concatenates one data set and uses it to input the model. Simple concatenation is insufficient to learn all domains efficiently.

As recorded in the previous literature, AE secures a better performance of feature extraction and selection. There have been various research studies related to feature selection and extraction; however, the accuracy of the learning models fluctuates because feature selection is based on specific metrics. Feature extraction via AE shows higher accuracy than using other dimension reduction methods. However, learning two models at the same time, feature extraction via AE and RUL prediction, increases model complexity unnecessarily. As a remedy to this issue, some studies predicted Hi directly through AE. Even in this case, the difference found between inputs and outputs appears to represent a problem in that the latent space cannot be trained efficiently. Therefore, the study based on constructing the two models into one model is insufficient. In this work, we present a study consisting of one model, along with latent spatial layer learning of AE. The conclusions that we were able to draw after reviewing the literature are as follows:

1. Feature sources need to be rich and diverse. Estimating the remaining useful lifetime requires multiple aspects of bearing sensor signals. As per existing literature, time series data, frequency data, and time–frequency data are employed for feature extraction as bearing signals are usually vibration, current, and acoustic noise.
2. Feature selection is required for reducing abnormal effects from the whole dataset. A disturbance in machine learning is caused by the series of data not following the learning direction of the entire dataset; this disturbance is due to noise or an event occurring after a very specific accident. Selecting features is critical to reducing the effect of such noise. Thus, ignoring useful data is often inevitable, since abandoning certain data needed for logical reasoning will not keep the consistency of estimating RUL.
3. Feature synthesis improves the possibility of finding hidden correlations of raw data. Both the mathematical approach and data-driven approach are used for evaluating correlations and reducing the data dimension.

From these conclusions, it appears that there exists an opportunity to create a new predictive maintenance algorithm for rotary parts.

From these conclusions, it appears that there is opportunity to motivate new predictive maintenance algorithm of rotary parts.

## 3. Feature Ensemble Autoencoder Architecture

**Figure 2:**
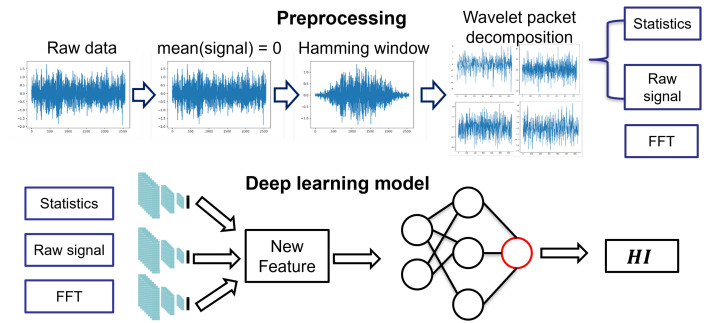
Procedure of proposed framework for estimating RUL.

Motivated by the opportunity mentioned in the previous section, we considered the improvement of feature extraction and selection as a tool to enhance the performance of RUL prediction. In this study, we propose a comprehensive data flow turned into an architecture incorporating ensemble learning with a feature ensemble autoencoder (FEAE), as depicted in Figure 2. The proposed model framework is designed to cover the following aspects:

1. WPD to distinguish low and high frequency in the time–frequency domain.
2. Application of an autoencoder to a multi-domain approach.
3. Reduction in multi-domain dimensions by using latent space generated by an autoencoder.
4. Application of a one-stage model with combined loss function to enhance the learning rate of the entire model.

A signal processing technique is first used to remove noise and redundant information from the signal. We then extract various domain features from time, frequency, and raw signal sources, and use them as input data for the FEAE model. The FEAE model consists of two parts. First, the FEAE learns latent space through each AE. At the same time, each latent space is generated through input of the RUL model, and finally, HI is predicted.

### 3.1. Signal Processing of Wavelet Packet Decomposition

Since the bearing vibration signal contains important information such as trouble and machine fault, accurate vibration analysis is an essential process. First, a direct component (DC) value removes from the signal the purpose of right transformation, such as Fourier and wavelet. Second, to remove noise in the signal, we apply a window function since the edge of the signal bears many uncertainties.

WPD is a time–frequency analysis method widely used in the field of signal processing We applied WPD to preprocessing of the proposed procedure to improve the resolution of complex signal data. In the case of WPD, the signal is decomposed into coefficients (low-frequency components) and details (high-frequency components) at the first level. This can be thought of as the low-frequency and high-frequency pass filter components of the signal [36].

The main difference between the wavelet transform (WT) and the WPD is the extent to which the signal is decomposed after the first level. Simply put, WPD is WT that passes more filters in signal processing. WT decomposes only low-frequency components at subsequent levels, while WPD decomposes low-frequency and high-frequency components at each level [37]. Therefore, WPD provides better resolution in various areas where the signal contains high-frequency information.

**Figure 3:**
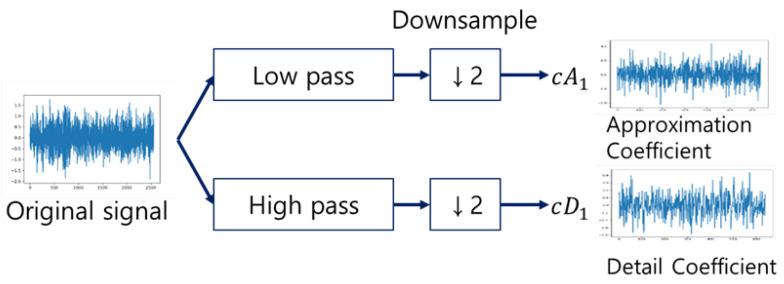
Procedure of discrete wavelet transformation.

The equation W (p, q) presented below is an equation for WT. x(t) is the signal to apply WT, ψ* (t) means a certain wavelet (mother wavelet), and ψ* means a complex conjugate. Furthermore, p plays the role of reducing or increasing a certain wavelet as a scaling parameter, and q means a variable that moves a certain wavelet as a movement parameter. The formula DWT (discrete wavelet transform) (I, j) can be obtained from the dioxide of W (p, q); p and q are replaced by 2i and 2j, respectively. Repeated decomposition can be used to obtain low-frequency (cA1) and high-frequency (cD1) band signals, as shown in Figure 3.

**Figure 4:**
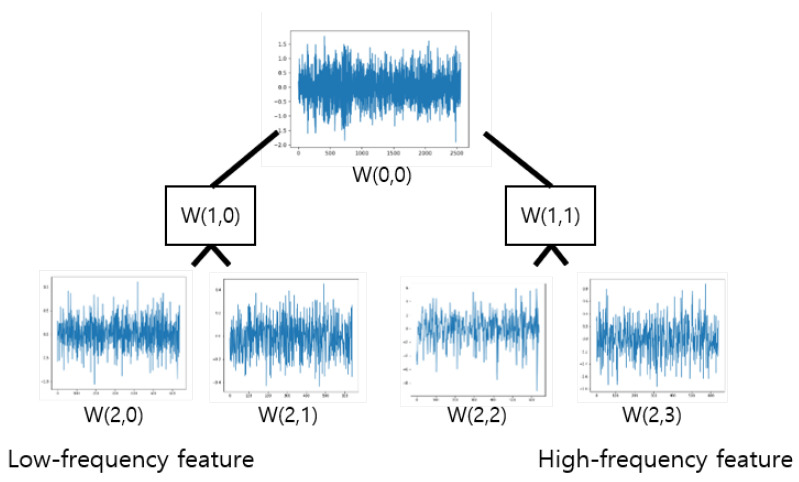
Decomposition result of WPD up to Level 2.

When the original signal is decomposed into Level 2 via the WPD process, it can be decomposed into a total of four signals, as shown in Figure 4. WT is considered to be a nonstationary signal that works as a frequency filter and is effective in reducing noise [38,39]. The decomposed signal contains low-frequency–high-frequency features. Next, in order to execute the preprocessing of the input data, the four decomposition signals and the original signal are created as one data set, allowing the preprocessing to be executed.

**Figure 5:**
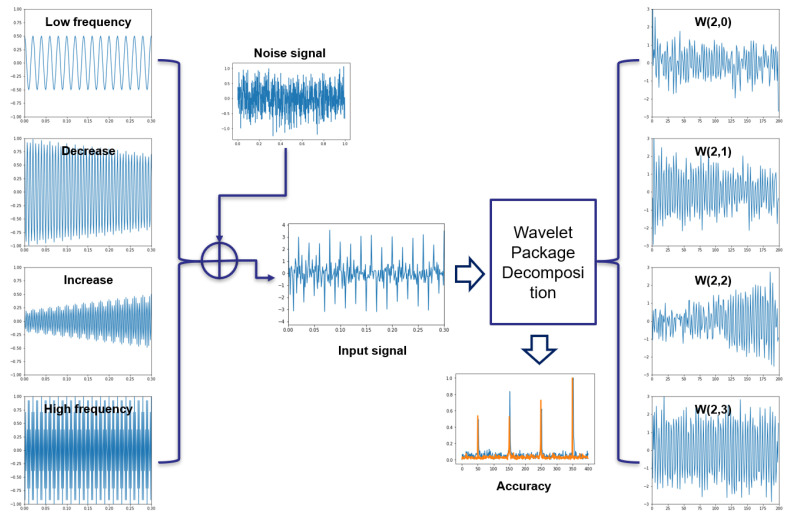
Resolution result of different mixed signals by using Level 2 WPD.

In particular, the resolution of WPD helps to detect a progressive increase in specific vibration caused by the growth of cracks in rotating components. Figure 5 shows the advantage of WPD in estimating the RUL. The input signal data are made by combining narrow, low signal, decreasing signal, increasing signal, wide high-frequency signal, and random noise signal in several different frequencies. The result of WPD maintains the trends of each signal, even though the noise signal is mixed. The accuracy of WPD’s resolution is evaluated with the result of the FFT filter and the DC-removing filter. Orange peaks cover the blue peaks of the original signal, as shown in Figure 5.

### 3.2. Preprocessing of Multi-Domain Datasets

In this section, we will focus on the way to extract feature sets. From raw signal, preprocessing is required to extract bearing degradation signal efficiency. Typically, feature sets of bearing data can be categorized into signal, statistics, and frequency. In the case of the time domain basis feature set, several traditional features were introduced for predicting the failures of bearings, such as RMS, kurtosis, and skewness. In the case of the frequency domain basis feature set, a Fourier transform that transforms the time domain vibration data into a frequency spectrum is used to extract frequency domain information. In the case of the time–frequency domain basis feature set, wavelet analysis is used to analyze the signal as a different resolution. In this study, we use three domain features that are discussed below:

1 Signal: Original signal and wavelet signal are used to signal the domain feature.
2 Statistics: Statistics of original signal and wavelet signal are used to statistically signal the domain feature.
3 Frequency: Unlike the above-mentioned features, the frequency feature only works with the original signal.

#### 3.2.1. Signal Feature Set

**Figure 6:**
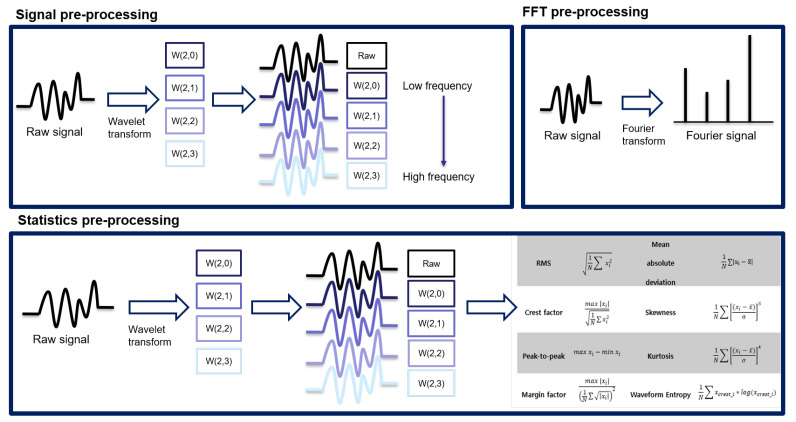
Preprocessing of each signal source.

The basic signal is an input type that is widely used in various studies that apply deep learning. This approach does not require preprocessing. Additionally, basic signals are used in the most common form to which the basic signal processing algorithms and WPD described in Section 3.2, are applied. As shown in Figure 6, in the final data set, it is used as one dataset to which the original signal and the wavelet are applied.

#### 3.2.2. Statistics Feature Set

This approach consists of extracting the statistics of the converted signal and the original signal using WPD. Statistics often used in RUL prediction are RMS, kurtosis, peak-to-peak, marginal factor, mean absolute deviation, skewness, and waveform entropy. A total of eight statistics are used. Since 8 statistics of the wavelet-transformed signal and the original signal are extracted, a total of 40 types of statistics is extracted. Therefore, it is possible to efficiently extract various information borne by each frequency signal. The overall procedure for the statistics feature set is shown in Figure 6.

Our proposed deep learning model essentially makes use of easily obtainable frequency domain signals at the input level. Time domain features are correlated with the overall trend for the bearing fault and constitute the fundamental inputs of the deep learning model for machine degradation. Because of the nature of raw data that is not found with frequency filters, primary statistics of raw data is selected as the first input of the proposed model; this also includes statistical figures directly obtained from the raw data and a method for collecting raw data characteristics, contrary to the frequency domain.

#### 3.2.3. Frequency Feature Set

FT has the advantage of being able to move signals from the time domain to the frequency domain. In addition, various signal frequencies can be decomposed of frequency via a periodic function in order to confirm the characteristics, as an intuitive understanding of FT. The following equation is a Fourier transform equation that decomposes a signal via a sine periodic function. Fourier transform plays a role in converting the signal from the time domain into the frequency domain. However, since the signal obtained via WPD is already a signal decomposed for each frequency, the same information will be obtained when FT is applied. For this reason, as shown in Figure 6, the WPD was not applied to frequency, which is a frequency domain feature set, and only FT was applied to the original signal.

### 3.3. Ensemble Learning of the Latent Space Generated by Autoencoders

In statistics and machine learning, ensemble methods use multiple learning algorithms to obtain a better predictive performance than that of any of the constituent learning algorithms taken alone. Unlike a statistical ensemble in statistical mechanics, which is usually infinite, a machine learning ensemble only consists of a concrete finite set of alternative models but typically represents a much more flexible structure among other alternatives. An ensemble model method is a machine learning process designed to obtain better prediction performance by strategically combining multiple learning algorithms. There are three primary advantages brought by ensemble methods. The first one is of a statistical nature and related to the lack of sufficient data to properly represent the data distribution. Without sufficient data, many hypotheses which give the same training accuracy may be chosen as the learning algorithm. Ensemble methods can thus reduce the risk of selecting the wrong model of aggregating all these candidate models. The second advantage is computational. Many learning algorithms, such as the decision tree and the neural network, operate by performing some form of local search. These methods frequently result in locally optimal solutions. Ensemble methods show clear advantages in this case, by running several local searches from different starting points. The last advantage is of a representational nature. In most cases, the true function, f, cannot be represented by any single hypothesis, H. However, the function can better be approximated by a weighted sum of several hypotheses.

### 3.4. One-Stage Learning Model for RUL Prediction

**Figure 7:**
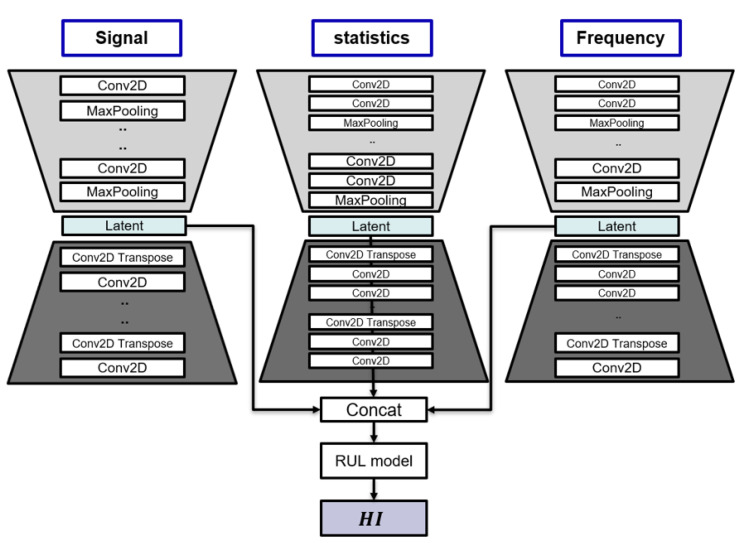
Network model of one-stage feature ensemble autoencoder for estimating RUL.

In the final analysis, all methods described in the methodology above are used to provide a new model, FEAE. Firstly, we proceed with the ensemble of input data. The structure shown in Figure 7 is used to efficiently compress and extract the information that each input data bears. This structure is a model based on three AEs, and each model uses a feature set of three regions as input values. Each feature set represents the input data of signal, statistics, and frequency, as described above. Additionally, the latent space of each AE is connected to the RUL model that predicts HI. The AE and RUL models consist of one stage, that is, one model.

There are two main models proposed: the first is the AE that learns latent space, and the second is a model that predicts HI using the latent space as an input to the RUL model. FEAE will eventually use the feature set of the three areas—signal, statistics, and frequency—as input. In addition, the final output has a total of four outputs with three AE restoration results for each feature set and one RUL model. The advantage of these models’ structures is that the latent space of AE is efficiently trained by two elements. First, the latent space is trained through the restoration loss function of the AE. Second, each latent space is used as an input in the RUL model and predicts HI. The latent space can be learned via mean square error (MSE), the loss function of this RUL model, which is advantageous.

### 3.5. Health Indicator of Remaining Useful Life

**Figure 8:**
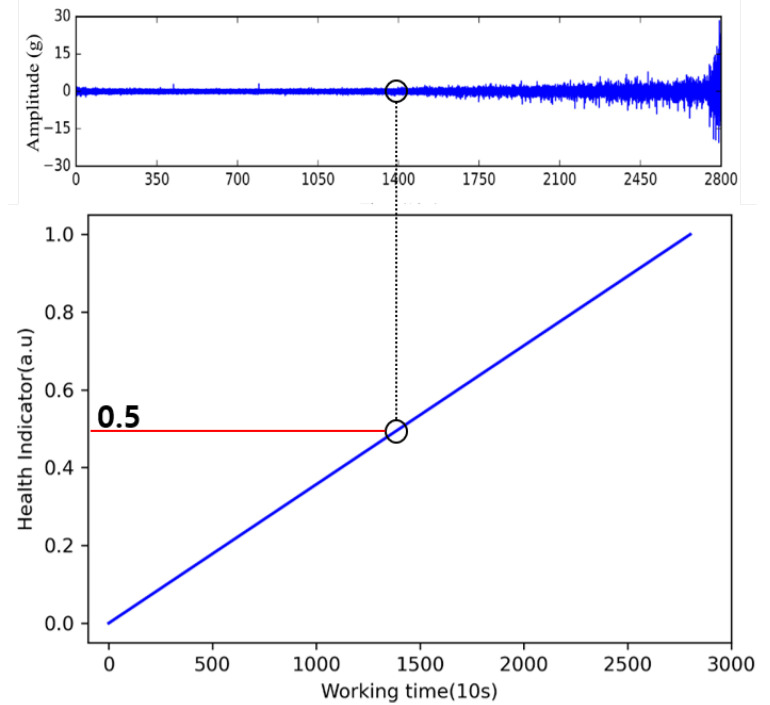
Definition of health indicator.

There are several ways in which HI can be designed. However, HI, which predicts the machine failure time, is generally highly volatile making it difficult to set a failure threshold. In general, even in the case of bearings, the threshold value is determined experimentally because the HI value is different for each work and data. To dispel these ambiguities, this study also uses common HI design methods. The HI value in the initial state is set to 0%, and when a complete failure occurs, the HI value is set to 100% in order to determine the HI value that can be applied for multiple situations. For example, if the total drive time is 2800 s and the current time is 1400 s, the current HI is set to 0.5, as shown in Figure 8.

(1) $$\left(x_{t},y_{t}\right),where\;t\in T,x_{t}\in F,\;\left(F=Feature\;set\right)$$

## 4. Experiment

### 4.1. Dataset

The data used in the experiment to validate the methodology is the IEEE PHM challenge PRONOSTIA dataset of 2012 [40]. The PRONOSTIA data consists of three working conditions, and the number of bearings for each experimental condition is seven, seven, and three, respectively. In addition, the sampling frequency of each bearing is 25.6 kHz, accelerometers are attached to the *x* and *y* axes, and the vibration of the two axes can be obtained. Finally, it consists of a run-to-failure dataset collected from the start of operation until the occurrence of corruption. The summary of data is shown in Table 2.

**Figure 9:**
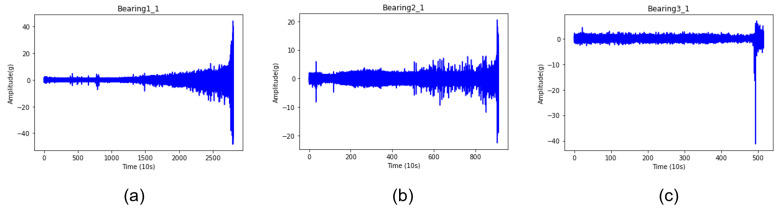
Measured vibration profile of test bearings: (**a**) Test Bearing1_1, (**b**) Test Bearing2_1, (**c**) Test Bearing3_1.

Three datasets are used as the test data required for methodological verification. Each dataset has a tendency, as shown in Figure 9. The detailed explanation is given in Table 3, as each has an individual working condition. Bearing1_1, Bearing2_1, and Bearing3_1 have increasingly complex failure patterns, along with common failure patterns.

### 4.2. Comparison

#### 4.2.1. Single-Input Models

**Figure 10:**
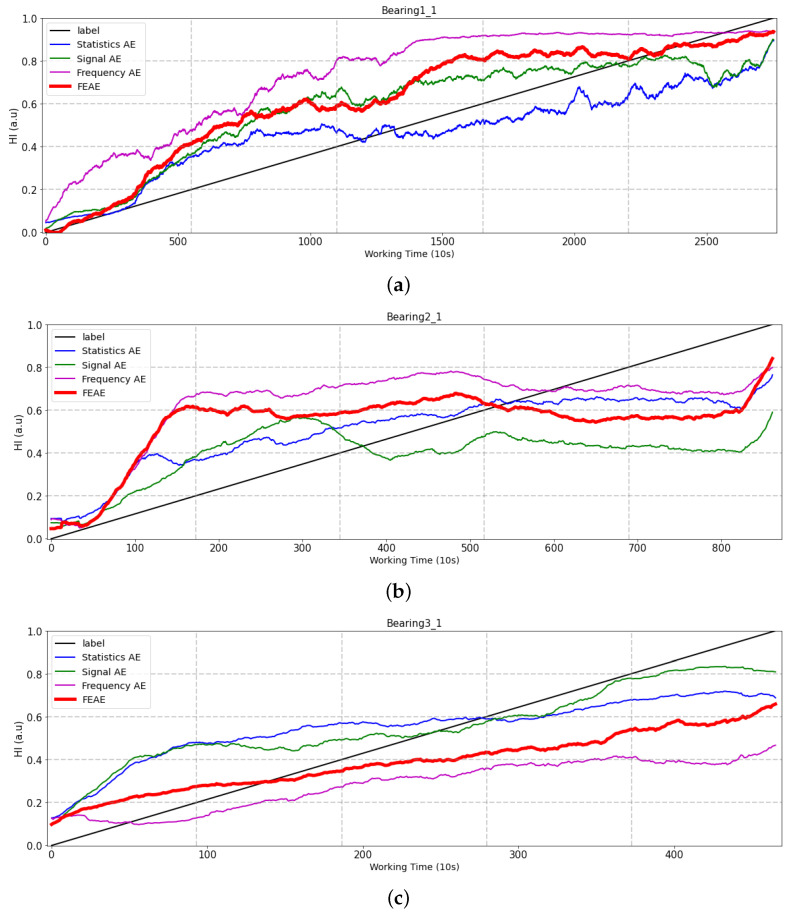
Comparison of experiment results of single-input models. (**a**) Test bearing1_1, (**b**) Test bearing2_1, (**c**) Test bearing3_1.

In this section, we compare the FEAE model with a single-input model. As shown in Figure 10, a comparison is made between the use of a single-input model and that of all input data. This is an experiment to evaluate the performance of the model structure.

1. Signal autoencoder;
2. Statistics autoencoder;
3. Frequency autoencoder.

1. Bearing1_1: This is the case of the simplest bearing failure pattern with a continuous amplitude increase. All models predict HI rather accurately, and the statistics AE predicts the HI closest to the label in a single-input model. However, in the case of the frequency AE model, there is a rapid amplitude increase, and some differences with other models can be detected. Finally, the signal AE model appears to be similar to the proposed FEAE model; however, after the second half, HI decreases again and shows unstable prediction.
2. Bearing2_1: With amplitude in the initial state, this is a case where amplitude continues until the latter half and observes a failure pattern after repeating momentary changes in amplitude. In the initial state, the statistics AE and the signal AE tend to increase in close proximity to the label, while the frequency AE and FEAE models tend to increase rapidly. In the middle state, signal AE tends to decrease suddenly, although the tendency is almost similar. In the latter half, in the cases of the statistics AE and the frequency AE, we can detect a pattern in which HI decreases again in the last part. This is a negative tendency, due to the characteristics of RUL. In addition, the proposed FEAE and signal AE models tend to increase until the end. In the final RUL part, the FEAE model shows the HI closest to the label and tends to increase until the end.
3. Bearing3_1: This dataset shows a pattern in which a value equal to or greater than the occurrence of a momentary change in amplitude is obtained after maintaining the amplitude in the initial state. The statistics AE and the signal AE show a downtrend in the last part, indicating bad predictions. The frequency AE and FEAE models tend to be closest to the label, showing a steadily increasing trend.

We indicate here below the calculated metric that evaluates HI for the three models.

In case of monotonicity, K is the number of data in the entire life cycle. Monotonicity = 1 indicates a complete monotonic character; in other cases, it means that HI oscillates. There is an irreversible relationship between the actual machine failure tendencies. Failure does not recover spontaneously without human intervention. Appropriate HI monotonicity tends to increase or decrease monotonically, which is usually the case.

Trendability shows a linear correlation between operating time and HI. K is the number of data in the entire life cycle, x means HI, and it represents the operating time. As the operating time increases, the fault gradually decreases. Therefore, for the trendability of the general situation, that is, the time and HI linear correlation, a value close to 1 will be a meaningful metric.

Criteria refers to the average value of both metrics. Rather than relying only on one of the two indicators, using the average of both values provides a more reliable indicator.

Summarizing the experiments on the three datasets, the statistics AE is performing well on average. Furthermore, in certain situations, it shows good performance when using a certain feature set. However, in the case of the FEAE model, it is found to maintain a stable tendency for any given situation, while having strong robustness. These characteristics are the result of efficiently learning the unique information of the vibration signal that is built for each time, frequency, and signal.

(2) $$Mon\left(X\right)=\frac{1}{N-1}\left|No.of\;\frac{d}{dx}>0-No.of\;\frac{d}{dx}<0\right|$$

(3) $$Corr\left(X,T\right)=\frac{\left|\sum_{i=1}^{N}\left(X_{0}^{i}-\bar{X}\right)\left(T^{i}-\bar{T}\right)\right|}{\sqrt{\sum_{i=1}^{N}\left(X_{0}^{i}-\bar{X}\right)\sum_{i=1}^{N}\left(T^{i}-\bar{T}\right)}}$$

(4) $$Cri=\frac{Corr+Mon}{2}$$

#### 4.2.2. Simple Concatenate Model

**Figure 11:**
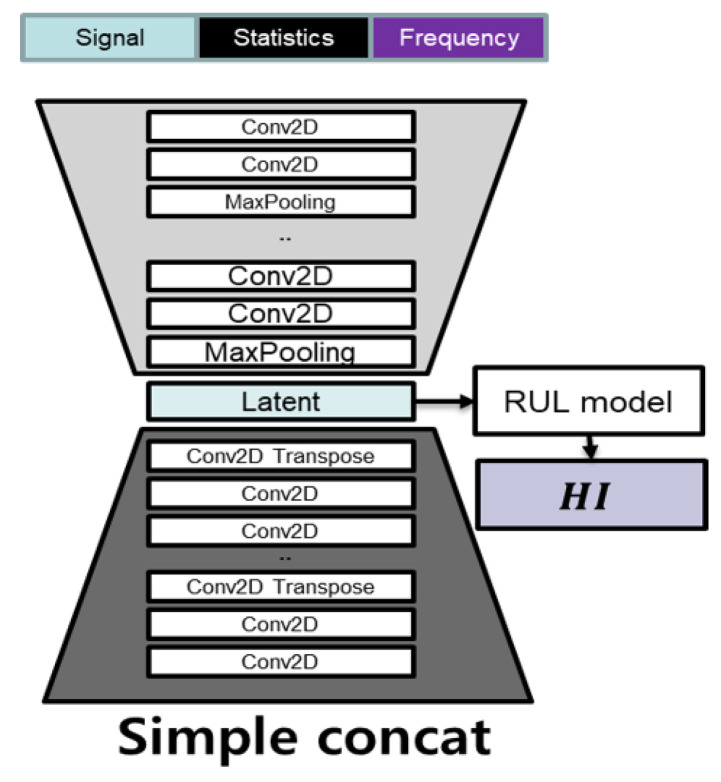
Network models of simple concatenate.

**Figure 12:**
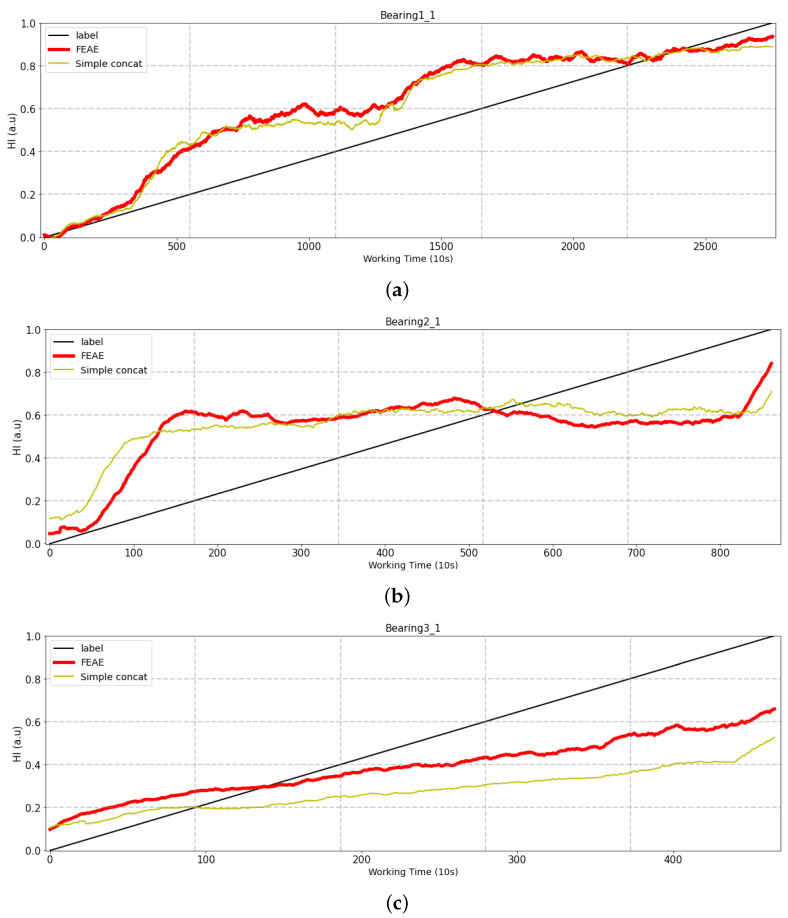
Comparison of experiment results of simple concatenate models. (**a**) Test bearing1_1, (**b**) Test bearing3_1, (**c**) Test bearing3_1.

The FEAE model uses all feature set combinations, and at the same time, is trained through individual AEs with signal, statistics, and frequency for each feature set having different meanings. We also use the same dataset and compare it with the basic simple concatenate model shown in Figure 11. The comparison of both models uses the same data, indicating whether the performance of the models changes depending on how they are trained. The results are comparison in Figure 12.

1. Bearing1_1: There is no large difference between the FEEA and simple concatenate models.
2. Bearing2_1: The same aspect is shown as in the case of Bearing1_1; however, there are a few differences. The simple concatenate model shows an increase in the initial state, whereas the FEAE model does not. It can also be seen that the FEAE model demonstrates even better predictive performance as regards the final HI predictive value.
3. Bearing3_1: There is a great difference in performance in comparison with the previous test bearing. Bearing3_1 represents data with outliers, and the two models differ greatly in terms of performance. Both models also show a steady rise, but the FEAE model can be seen to perform better.

The difference between the performance of the two models changes depending on how the input data are trained. The simple concatenate model and the FEAE model learn the same data. The FEAE model trains the features of statistics, signals, and frequency domains through different AEs for each domain. In contrast, the simple concatenate model concatenates three domains into a single dataset and uses the merged dataset as the training data. The difference between these learning methods resides in the performance of these models. The method of learning features as one dataset as reported in existing research is not able to efficiently extract the unique information of each domain that each source has.

#### 4.2.3. Number of Stages

**Figure 13:**
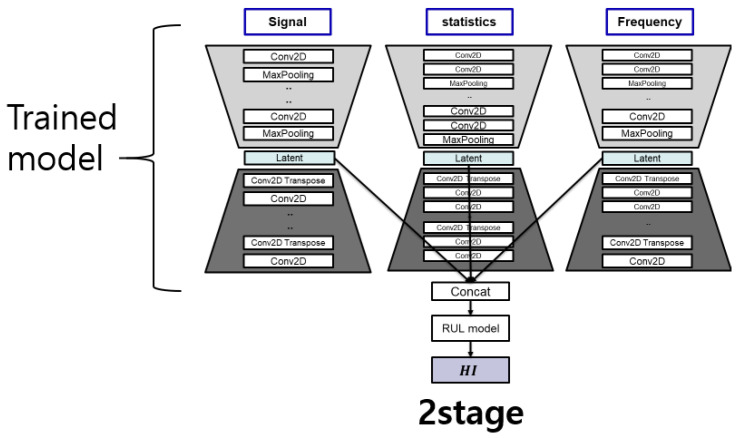
Network models of two-stage FEAE.

**Figure 14:**
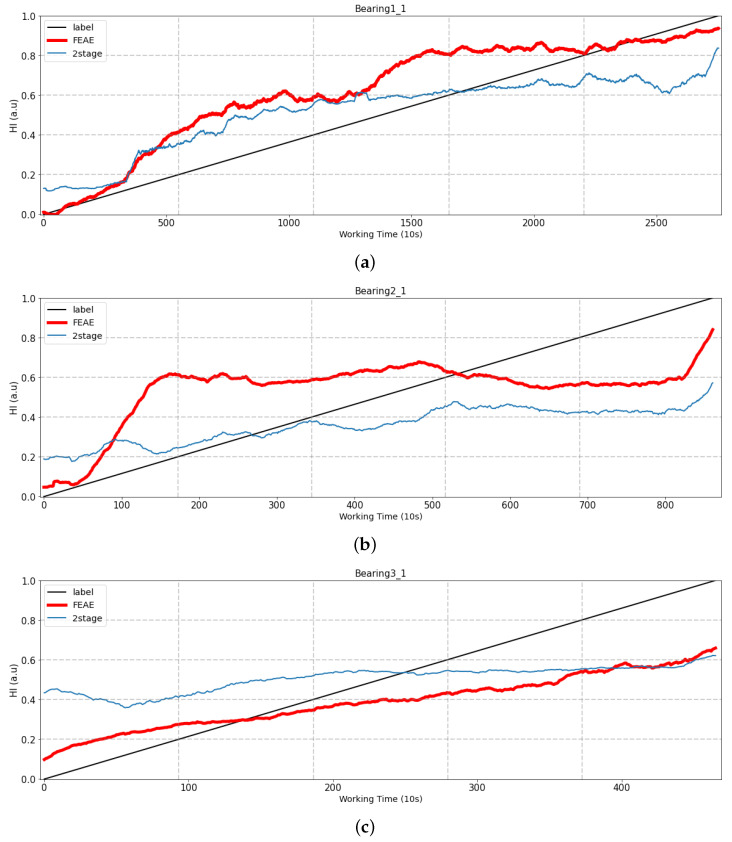
Comparison of experiment results of multi-/single-stage models. (**a**) Test bearing1_1, (**b**) Test bearing2_1, (**c**) Test bearing3_1.

The FEAE model (one-stage model) and the two models (two-stage model) that extract features through AE and prediction of HI are compared in this section. The difference between the one-stage model (FEAE) and the two-stage model is that learning is performed at the same time, features are extracted using the AE that has been trained, and the RUL model is trained. This experiment is comparative and conducted to learn how the latent space can be learned efficiently by the two loss functions. We also used the same dataset and compared it with multi-/single-stage models, as shown in Figure 13. The results are comparison in Figure 14.

1. Bearing1_1: It looks similar to the initial state, but as time goes on, the two-stage model shows that HI is no longer increasing.
2. Bearing2_1 and Bearing3_1: Two experiments show that the initial HI start is non-zero. This can be seen as a result of the vibration signals of the two bearings being generated with the initial amplitude maintained. In addition, the two-stage model does not show a large increase over time. These features indicate that the latent space of the trained AE does not act as an efficient input feature, and discussions related to training the RUL prediction model are to be conducted.

Learning the RUL prediction model using the latent space of trained AE as an input feature is common to both models (one stage and two stages). However, the difference between both models is that, when using a trained AE, the AE and the RUL prediction models are trained. In the case of the one-stage model (FEAE), the latent space is trained by two loss functions that are the loss functions of the AE and RUL prediction models, respectively. The two loss functions efficiently learn the latent space of each AE through the backpropagation method, which is a deep learning method, and at the same time, the final output of the model has better prediction performance of HI. However, in the case of the two-stage model, the models that predict AE and HI performing feature extraction are expected to show poor performance, since they are trained separately.

#### 4.2.4. RNN-Based Models

**Figure 15:**
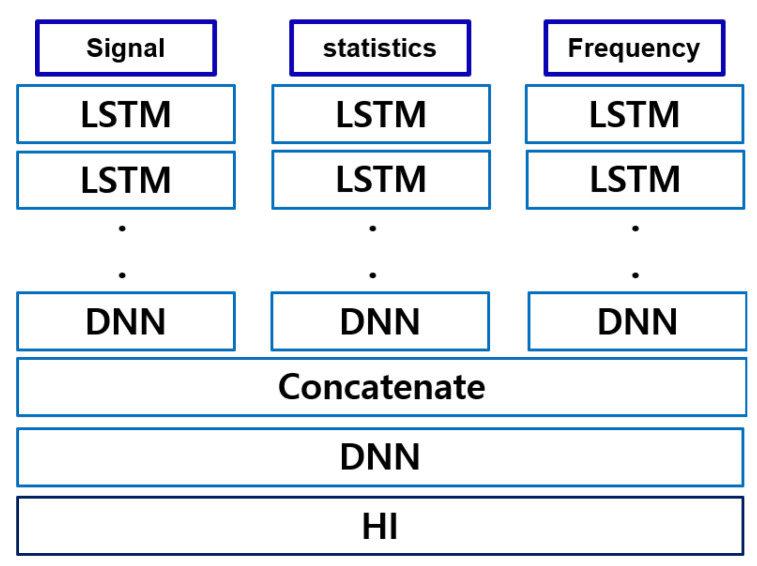
Network models of one-stage FEAE and RNN.

**Figure 16:**
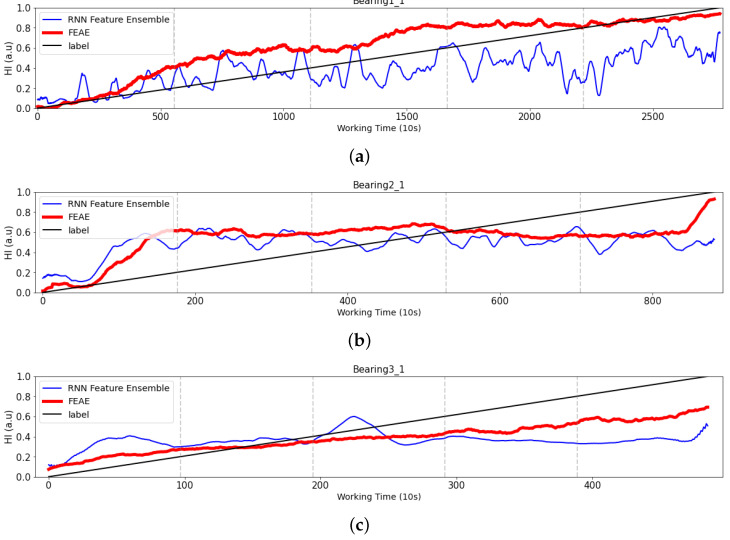
Comparison of experiment results of RNN type models. (**a**) Test bearing1_1, (**b**) Test bearing2_1, (**c**) Test bearing3_1.

Finally, a comparison was made between the AE-based model and the RNN-based model. RNN-based models have been traditionally used to handle time series and are among classic methods used in the literature. Our experiment describes the difference between a model that uses the latent space of AE and a model that predicts HI using an existing RNN-based model. We also used the same dataset and compared it with RNN-type models, as shown in Figure 15. The results are comparison in Figure 16.

1. Bearing1_1: In the case of the RNN model, there is a continuous oscillation compared to the FEAE model. A continuously increasing trend is observed until the second half. However, as it progresses, there is a decreasing tendency, which is then followed by an increase.
2. Bearing2_1: The RNN model starts at 0.2 and is similar to the FEAE until it approaches the second half. However, it can be seen that the FEAE model increases in the part that predicts the final HI, whereas the RNN model does not increase, but predicts a constant HI.
3. Bearing3_1: In the initial state, the RNN model and the FEAE model have the same tendency, but it can be seen that the RNN model does not rise with time as it goes beyond the middle state. Finally, the RNN model can be seen to slightly increase at the end.

In the cases of the FEAE model and the RNN model, the difference resides in that they are an AE-based model and an RNN-based model, respectively. A model using the latent space of AE can demonstrate higher performance than an RNN model using the data of each domain as a feature, without performing feature extraction. We can also observe that the FEAE model increases in a more stable manner than the RNN model.

#### 4.2.5. Comparison with Other Learning Models

This section compares the proposed one-stage FEAE model with previously developed model using three different types of failure bearings. The final health indicator, monotonicity, trendability, and critical factor were used as comparison criteria. The important indicators of failure diagnosis are the reliable information about the monotonicity and the final health indicator. The final health indicator is very important when determining whether machine stops now or not. In particular, the final health indicator is used to set the threshold value of the failure decision that is recognized as a breakdown. Secondly, the monotonicity is also a critical factor because if the health indicator rises too high before its breakage the prediction algorithm alerts too early and losses the remaining life of the unbroken bearing.

**Figure 17:**
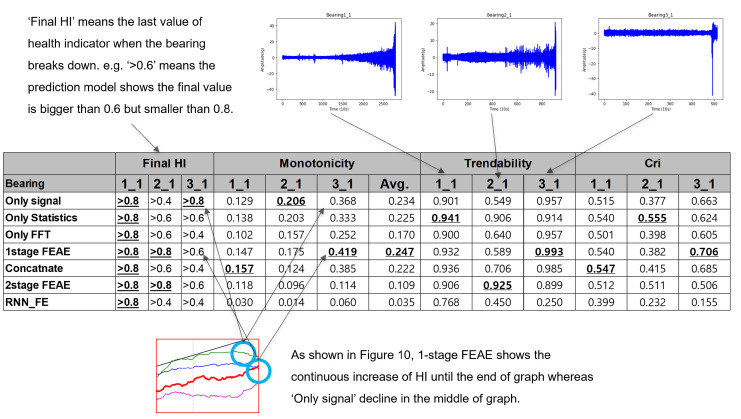
Performance comparison of the proposed method and previous approaches.

As shown in Figure 17, as a result of comparing the seven models, the health indicators of the first bearing, which are constantly damaged, all reached 0.8 or higher. On the other hand, in the case of the second bearing, abnormal vibration occurred in the middle, so it was difficult to accurately detect when only single feature was used, and a high health indicator was measured when the proposed FEAE was used. Finally, the third is a case in which the breakage occurred suddenly in the final stage, and only the model using only raw signal data showed its health indicator was close to 0.8. However, in the case of using the raw signal, a low score appeared in the monotonicity because the health indicator fell down in the final stage. This phenomenon is recognized as a failure before its actual breakdown. It means that the bearing cannot be used until a sufficient end life. In terms of monotonicity, in the comparison of the average of the three bearings, FEAE shows the highest scores and received overall good scores in trendability and critical factor scores too.

Comprehensively, even though the proposed FEAE did not receive the highest scores in all criteria, it received excellent scores overall. In particular, the proposed model was excellent in the final health indicator and monotonicity, which are the most important indicators of failure diagnosis. Therefore, it can be said that the proposed method shows stable performance in terms of the fault diagnosis algorithm.

## 5. Conclusions

In this study, we have proposed a methodology to solve the problems related to the selection and extraction of input data and features that should be considered in bearing life prediction. To solve the problem of input data, the signal processing technique classically used for vibration signal analysis was applied. In addition, vibration signals divided each frequency band via WPD, and each domain was preprocessed to propose a method used in various studies. We applied feature extraction through AE to solve the feature selection problem. By integrating the two-stage model, which is a drawback of the model using AE, into the one-stage model, we were able to resolve the complicated issue associated with the use of the two models, while obtaining a model with high performance.

Experiments were performed using the PRONOSTIA dataset to verify this methodology. Through the FEAE model presented in this study, it was shown that higher performance was obtained than that of the model using a single AE. This FEAE model made it possible to learn the meaning of the feature more efficiently than is the case when the functions of each area were created as one data set. In addition, feature extraction through AE was shown to be efficient. By converting the two-stage model into the one-stage model, we were able to obtain a model with even better performance.

There are various facilities and working conditions on a given site. However, there are many difficulties in applying one algorithm to each facility. If there exists an area that best describes each facility, and if the working conditions change, then the above-mentioned problems can occur. However, when applied to equipment via the proposed method, we can design models that are stronger or have better performance through ensembles in various areas. When predicting the remaining life, based on these advantages, field workers can obtain more reliable results.

Research for predictive maintenance of bearings is the main source of prediction of remaining life. However, future research should focus on analyzing the value of HI calculated by any factor through explainable artificial intelligence. As the first step in this process, it will be necessary to analyze the latent space of AE. In the next stage, additional information will have to be obtained from the data, investigating the relationship between each input function and the latent space, while properly interpreting the HI value. Otherwise, to improve the efficiency of the learning model, future works should consider the application of a low-complexity CNN network [39,40] or transfer learning to other dataset [41,42].

## Figures and Tables

**Table 1:** The comparison of inputs, features, and models of key papers.

Paper	Input	Feature	Model
Guo, L. et al. (2017) [14]	Statistics FFT Wavelet	Selection	RNN
Ren, L. et al. (2018) [7]	Statistics FSPS Wavelet	Extraction only statistics /Concatenate	AE + MLP
Zhu, J. et al. (2018) [15]	Wavelet	X	CNN
Wang, B. et al. (2019) [16]	Raw signal	X	CNN
Li, X. et al. (2019) [17]	STFT	X	CNN
Lin, P. et al. (2019) [18]	FFT	Extraction	AE + MLP
Sadoughi, M. et al. (2019) [19]	Statistics	Selection/Extraction	AE + LSTM
Ren, L. et al. (2019) [20]	Statistics FFT Wavelet	Concatenate	GRU
Wang, H. et al. (2020) [21]	Raw signal	Extraction	AE + LSTM

**Table 2:** Measurement conditions.

Experiment Condition	Number of Bearings	Notation	Experiment Information
1800 RPM and 4000 N	7	Bearing1_1 bearing1_7	sampling period interval: 10 s
1800 RPM and 4000 N	7	Bearing1_1 bearing1_7	sampling frequency: 25.6 KHz
1800 RPM and 4000 N	7	Bearing1_1 bearing1_7	0.1 s snapshot, x, y axis accelerometer

**Table 3:** Breakdown conditions.

Bearing	Description
Bearing1_1	general failure pattern, monotonic increasing amplitude
Bearing2_1	general failure pattern, sudden increasing amplitude
Bearing3_1	general failure pattern, sudden increasing amplitude, outlier

## Abbreviations

**Abbreviation**	**Definition**
PHM	prognostics and health management
PdM	predictive maintenance
RUL	remaining useful life
HI	health indicator
WT	wavelet transformation
DWT	discrete wavelet transformation
FT	Fourier transformation
WPD	wavelet packet decomposition
AE	autoencoder
FEAE	feature ensemble autoencoder
